# p53 immunoreaction in endoscopic biopsy specimens of colorectal cancer, and its prognostic significance.

**DOI:** 10.1038/bjc.1993.348

**Published:** 1993-08

**Authors:** A. Yamaguchi, G. Nakagawara, Y. Kurosaka, G. Nishimura, Y. Yonemura, I. Miyazaki

**Affiliations:** First Department of Surgery, Fukui Medical School, Japan.

## Abstract

**Images:**


Br. J. Cancer (1993), 68, 399-402 © Macmillan Press Ltd., 1993

### p53 immunoreaction in endoscopic biopsy specimens of colorectal cancer, and its prognostic significance

A. Yamaguchi[1], G. Nakagawara[1], Y. Kurosaka[2], G. Nishimura[2], Y. Yonemura[2] & I. Miyazaki[2]

[1]*First Department of Surgery, Fukui Medical School;* [2]*Second Department of Surgery, School of Medicine, Kanazawa University, Japan.*

**Summary** The expression of p53 protein was immunohistochemically studied in formalin-fixed paraffin-embedded biopsy specimens of 203 colorectal carcinomas by use of a monoclonal antibody specific for the p53 protein, PAb1801. p53 protein expression with its reactivity localised in nuclei was found in 121 (59.6%) of the cancers. There was no correlation of p53 immunoreactivity with histological classification, wall invasion, lymphatic invasion, venous invasion, lymph node metastases, or peritoneal metastases. p53-positive cancers were more frequently associated with liver metastasis than p53-negative ones. Patients with p53-positive tumours had significantly poorer prognoses than those with p53-negative tumours. The 5 year survival rate was 58.1% for patients with p53-positive tumours, and 76.3% for those with p53-negative tumours. In Dukes' stage C tumours, an especially good correlation was found between p53 immunoreactivity and prognosis. In addition, patients with p53-positive tumours had higher recurrence rates. The results indicate that p53 immunoreactivity may be a useful prognostic marker of colorectal cancers.

Germline or somatic inactivation of tumour suppressor genes through point mutation or deletion plays an important role in carcinogenesis. p53, one of these tumour suppressor genes, is a cellular protein discovered through its association with DNA virus antigen in murine cells (Lane & Crawford, 1979). This protein may be localised in different cellular compartments in normal and in transformed cells (Rotter *et al.*, 1983). The p53 protein in normal cells, or p53 of the wild type, restrains cell growth, and probably acts as a tumour suppressor (Fearon & Vogelstein, 1990; Lane & Benchimol, 1990, Levine, 1990). Crawford *et al.* (1981) reported that many human tumour-derived cell lines show elevated levels of p53 protein. In addition, the p53 protein in tumour-derived cell is explained as the mutated forms of p53 (Lane & Benchimol, 1990). Also, a mutant p53 with an extended half-life induces progression of growth-arrested, resting cells to an active, cycling state, and causes immortalisation of normal cells and their malignant transformation in cooperation with H-*ras* oncogene (Mercer *et al.*, 1982, Reich & Levine, 1984; Parada, 1984). p53 expression has been reported in a number of human tumours, including those of the breast, lung and colorectum (Crawford, 1984; Cattoretti, 1988; Iggo, 1990). Several authors have recently shown the relationship between p53 overexpression and prognosis in breast cancer (Cattoretti *et al.*, 1988; Iwaya *et al.*, 1991). In this study, we analysed 203 colorectal cancers immunohistochemically, using the PAb1801 mouse anti-p53 MAb, and found that p53 immunoreactivity is correlated with clinicopathologic data and prognosis. Also, we report herein the relationship between p53 overexpression and the DNA ploidy pattern.

### Materials and methods

We investigated the materials from 203 colorectal cancer patients (112 colon, and 91 rectal cancer patients) who had undergone resection of the malignancies in the Second Department of Surgery, Kanazawa University. Carcinomas were reviewed and graded by a single pathologist using criteria recommended by the general rules of clinical and pathological studies on cancer of colon, rectum and anus for histological type, depth of tumour invasion, lymphatic invasion and venous invasion (Japanese Research Society for Cancer of Colon and Rectum, 1983). Seventy-eight of the tumours were histologically diagnosed as well differentiated, 111 as moderately differentiated, five as poorly differentiated adenocarcinomas, and the other nine as mucinous carcinomas. Liver metastases were found in 44 (21.7%), peritoneal metastases in 16 (7.9%), and lymph node metastases in 98 (48.3%) of the patients. Twenty-five patients had stage A; 68, stage B; 55, stage C, and 55, stage D cancers by the Dukes classification.

The biopsy and resected specimens of the 203 lesions were fixed in 10% formalin overnight and embedded in paraffin. Thirty-nine surgically resected specimens were fixed by the AMeX (acetone, methyl benzoate, and Xylene) method (Sato *et al.*, 1986) and embedded in paraffin. Their sections were dewaxed, and the endogenous peroxidase activity was blocked by incubation of the sections in 1% hydrogen peroxidase in methanol for 30 min. The hydrated sections were incubated in a 1:5 dilution of normal goat serum at room temperature for 15 min to reduce non-specific staining, and incubated with a 1:20 dilution of primary monoclonal antibody PAb1801 (NOVOCASTRA Labo., Newcastle University) at room temperature for 2 h. After washing with Tris Buffer saline (TBS), the slides were incubated with a 1:30 dilution of biotinylated goat anti-mouse immunoglobulin G (TAGO INC, Burlingame CA, USA) at room temperature for 30 min, followed by incubation with a 1:100 dilution of streptoavidin-biotin-peroxidase complex (DAKO Patts) at room temperature for 30 min. The peroxidase activity was developed with 3-3'-diaminobenzidine tetrahydrochloride (DAB). Finally, the slides were stained with methyl green for 10 min. Negative control studies were carried out in the absence of the primary antiserum to p53. p53 staining was considered positive when neoplastic cells were clearly stained in the nucleus.

Thirty-micron-thick paraffin sections were deparaffinised with xylene, and then dehydrated in ethanols. The specimens were washed with distilled water and incubated in a 0.5% pepsin solution (Sigma Chemical Co., St Louis, MO, USA) at 37°C for 30 min. Nuclei were then filtered through a 37 $\mu m$ nylon mesh, washed twice with RPMI 1640 (GIBCO, Grand Island, NY, USA), and centrifuged. The remaining pellet was incubated in Hanks' solution containing 0.2% EDTA and 0.01% ribonuclease (Sigma Chemical Co., St Louis, MO, USA) at 37°C for 20 min, then centrifuged, resuspended in 1.0 ml of propidium iodide (50 $\mu g\ ml^{-1}$; Sigma Chemical Co., St Louis, MO, USA) in TBS, and incubated in the dark at 4°C for 1 h. About 20,000 cells were analysed with an EPICS

Correspondence: A. Yamaguchi, First Department of Surgery, Fukui Medical School, 23-3 Shimoaizuki, Matsuoka-cho, Yoshida-gun, Fukui 910-11, Japan.
Received 8 July 1992; and in revised form 8 December 1992.

**Figure 1** Immunohistochemical staining of p53 using a monoclonal antibody PAb1801. Nuclear staining is positive in almost all cancer cells.

C flow cytometer. A tumour with a single G0/1 was considered diploid, and diploid tumours were assigned a DNA index of 1.00. An additional abnormal G1 peak indicated the presence of aneuploid.

Statistical analyses of data were performed by the chi-square or Student's *t*-test. The outcomes from different groups of patients were compared by the generalised Wilcoxon test.

### Results

There were 121 cancers (59.6%) having the evidence of p53 protein overexpression with the reaction localised in the nuclei. Reaction-positive cells were distributed in almost all the cancer cells, but never demonstrated in normal colorectal mucosa (Figure 1). In 39 of the patients, p53 immunoreactivity could be determined in both formalin-fixed paraffin-embedded biopsy specimens and AMeX fixed paraffin-embedded resected ones; and 35 (89.8%) of them had the same pattern of p53 immunoreactivity in both specimens. More precisely, 23 of the patients had p53-positive tumours and 12, p53-negative tumours in both resected and biopsy specimens, while the other four (10.2%) differed in p53 immunoreactivity: three had p53-negative biopsy specimens and p53-positive resected specimens, and another, p53-positive biopsy specimens and p53-negative resected specimens (Table I).

The relationship between p53 protein expression and clinicopathological findings is shown in Table II. There was no correlation between p53 protein expression and histological classification, wall invasion, lymphatic invasion, venous invasion, lymph node metastases, or peritoneal metastases. The p53 protein-positive rate was 56.0% for 159 tumours without liver metastasis, and 72.7% for 44 tumours with liver

**Table I** Correlation between p53 immunoreactivity of endoscopic biopsy and that of resected specimens. There is a relatively good correlation between two groups

| *Resected specimens (AMeX)* | *Biopsied specimens (paraffin)* *p53 (+)* | *p53 (−)* |
|---|---|---|
| p53 (+) | 12 (30.8%) | 1 (2.6%) |
| p53 (−) | 3 (7.6%) | 23 (59.0%) |

**Table II** The relationship between p53 protein expression and clinicopathological findings

| *Viables* | *No. of cases* | *p53 (+)* | *Cases (%)* | |
|---|---|---|---|---|
| *Location* | | | | |
| Colon | 112 | 67 | (59.8%) | NS |
| Rectum | 91 | 54 | (59.3%) | |
| *Histological type* | | | | |
| Well differentiated | 78 | 46 | (59.0%) | NS |
| Moderately differentiated | 111 | 70 | (63.1%) | |
| Poorly differentiated | 5 | 3 | (60.0%) | |
| Mucinous | 9 | 2 | (22.2%) | |
| *Depth of tumour invasion* | | | | |
| m, sm | 8 | 4 | (50.0%) | NS |
| pm | 22 | 13 | (59.1%) | |
| ss | 106 | 63 | (59.4%) | |
| s | 53 | 33 | (62.3%) | |
| si | 14 | 8 | (57.1%) | |
| *Lymphatic invasion* | | | | |
| Negative | 44 | 23 | (52.3%) | NS |
| Positive | 159 | 98 | (61.6%) | |
| *Venous invasion* | | | | |
| Negative | 118 | 65 | (55.1%) | NS |
| Positive | 85 | 56 | (65.9%) | |
| *Lymph node metastasis* | | | | |
| Negative | 105 | 61 | (58.1%) | NS |
| Positive | 98 | 60 | (61.2%) | |
| *Hepatic metastasis* | | | | |
| Negative | 159 | 89 | (56.0%) | $P < 0.05$ |
| Positive | 44 | 32 | (72.7%) | |
| *Peritoneal metastasis* | | | | |
| Negative | 187 | 110 | (58.8%) | NS |
| Positive | 16 | 11 | (68.8%) | |

m, mucosa; sm, submucosa; pm, propia muscle; ss, subserosa, s, serosa; si, infiltration of serosa.

metastasis. There was a significant difference in the rate of liver metastasis between these two groups ($P<0.05$). By Dukes' staging, the p53 protein-positive rate was 68% for Dukes' stage A, 57.4% for Dukes' stage B, 47.3% for Dukes' stage C and 70.9% for Dukes' stage D tumours (Table III). There was no significant correlation between Dukes' stages and p53 immunoreactivity.

Figure 2 depicts the Kaplan-Meier curves for the survival of these two groups of patients. The 5-year survival rate was 76.3% for 82 patients with p53-negative tumours. However, the prognosis was poor in 121 patients with p53-positive tumours, and the 5-year survival rate was 58.1% for this group. The difference between these two groups was statistically significant ($P<0.05$). Among the patients with Dukes' stage A and B tumours, no difference was found in survival between the patients with p53-positive and those with p53-negative tumours, with the 5-year survival rates of 96.5% and 95.5%. Furthermore, there was no significant difference between the survival rates and the patterns of p53 immunoreactivity in patients with Dukes' stage D tumours. Among the 55 patients with Dukes' stage C tumours, however, those with p53-negative tumours survived significantly longer than those with p53-positive tumours ($P<0.05$) (Figure 3). The 5-year survival rates were 59.2% for 26 patients with p53-positive tumours, and 88.9% for those with p53-negative tumours. In 148 patients who underwent curative resection we examined the relationship between recurrence rate and p53 immunoreactivity. The patients with p53-negative tumours gave a recurrence rate of 7.6%, while those with p53-positive tumours recorded a recurrence rate of 18.3% the difference between the two groups was statistically significant ($P<0.05$).

In flow cytometric analysis, DNA diploidy (diploid) was detected in 52 (40.3%) of 129 patients, and 77 (59.7%) of the patients had tumours with DNA aneuploidy containing an abnormal DNA stemline (aneuploid). Forty-nine patients with p53-negative tumours consisted of 21 with diploid and 28 with aneuploid tumours, while 80 patients with p53-positive tumours were composed of 31 with diploid and 49 with aneuploid tumours. There was no correlation between the p53 immunoreactivity and the DNA ploidy pattern (Table IV).

**Table III** p53 immunoreactivity patterns and Dukes' stages. There is no correlation between p53 immunoreactivity patterns and Dukes' stages

| *Dukes' stage* | *No. of cases* | *p53 (+)* | *Cases (%)* |
|---|---|---|---|
| A | 25 | 17 | (68.0%) |
| B | 68 | 39 | (57.4%) |
| C | 55 | 26 | (47.3%) |
| D | 55 | 39 | (70.9%) |

NS

**Figure 2** Survival curves for all patients with colorectal carcinoma, classed by p53 immunoreactivity. Patients with p53-positive tumour run significantly poorer prognoses than those with p53-negative tumours.

**Figure 3** Survival curves for patients with Dukes' stage C tumour. Five-year survival rates are 88.9% for 29 patients with p53-negative tumour, and 59.2% for patients with p53-positive tumour: the difference between the two groups of patients is statistically significant.

**Table IV** Correlation between p53 immunoreactivity and DNA ploidy patterns

| | *DNA ploidy pattern* | |
|---|---|---|
| *p53 immunoreactivity* | *Diploid* | *Aneuploid* |
| (−) | 21 (42.9%) | 28 (57.1%) |
| (+) | 31 (38.8%) | 49 (61.3%) |

NS

### Discussion

We analysed the p53 protein expression immunohistochemically in colorectal cancers, using the PAb1801 mouse anti-p53 MAb. PAb1801 is a monoclonal antibody to human p53 protein that recognises a denaturation-resistant epitope between the amino acids 32 and 79 (Banks *et al.*, 1986). PAb1801 is reactive on both the wild type and mutant forms of p53. However, the level of wild type p53 is low, with a short half-life (Oren *et al.*, 1981; Reich *et al.*, 1983). On the other hand, the level of mutant p53 is 100-fold higher than that of the wild type p53, with a long half-life. In our study p53 immunoreactivity with PAb1801 was localised in the nuclei of cancer cells, and normal epithelia adjacent to cancers were completely negative for p53. Consequently, we have come to think that PAb1801 detects only the mutant p53 protein in formalin-fixed paraffin-embedded sections.

We compared the p53 immunoreactivity of formalin-fixed biopsy specimens with that of AMeX-fixed resected specimens. Thirty-five (89.8%) of the 39 suitably studied patients proved to have the same p53 immunoreactivity pattern in both specimens. p53 immunoreactivity was found in 121 (59.6%) of the 203 lesions, and was mainly nuclear, irrespective of fixation. Several investigators have reported the presence of p53 reactivity in colorectal cancers. Kawasaki *et al.* (1992), using microwave-fixed, paraffin-embedded sections, reported that nuclear p53 was detected in 51 (60.7%) of 84 colorectal cancers. We therefore concluded that it would be possible by use of PAb1801 to analyse the p53 immunoreactivity in formalin-fixed biopsy specimens preoperatively.

Recent studies have shown a close correlation between p53 immunoreactivity and prognosis of several malignant tumours. Cattoretti *et al.* (1988) demonstrated that p53 expression is associated with presence of the oestrogen receptor, the growth factor receptor, and the proliferation-associated antigen Ki-67 in breast cancer. Iwaya *et al.* (1991) reported that p53 protein immunoreactivity is a clinically useful indicator of breast cancer aggressiveness. We previously reported that immunoreactivity of p53 is an independent prognostic indicator of colorectal cancer (Yamaguchi, 1992). In addition, Kern *et al.* (1989) reported that deletion of 17p and that of 18q were significantly associated with distant metastasis and prognosis. Knowing the malignant potential preoperatively is important in choosing an adequate therapeutic method and in prognosis. We have therefore studied p53 immunoreactivity in endoscopic biopsies of colorectal cancer. There was no significant correlation between p53 immunoreactivity and histological classification, wall invasion, lymphatic invasion, venous invasion, lymph node metastases, or peritoneal metastases. Previous immunohistochemical studies of p53 described no significant correlation between p53 expression and site, differentiation, or Dukes' stage (Purdie, 1991). In our study, however, the patients with p53-positive tumours had a higher rate of liver metastases. In addition, p53 immunoreactivity showed a close inverse correlation with prognosis. By Dukes' staging, the 5-year survival rate of patients with Dukes' stage A and B tumour was over 90%, while the prognosis was very poor in patients with Dukes' stage D. Therefore, there was no significant correlation between p53 immunoreactivity and prognosis in Dukes' A, B and D cases. However among the patients with Dukes' stage C cancers, survival was significantly shorter when the tumour was p53-positive. Furthermore, the patients with p53-positive tumours had a greater relative risk of recurrence.

In general, aneuploid tumours are associated with poor prognosis (Wolley *et al.*, 1982; Armitage *et al.*, 1985). Here, however, we show no correlation between p53 immunoreactivity and DNA ploidy an observation similar to that of Scott *et al.* (1991). However, we previously reported that the growth fraction of p53-positive tumours is significantly higher than that of p53-negative tumours (Yamaguchi, 1992). Also, we argued that the detection of growth fraction by use of a monoclonal antibody against DNA polymerase α enables the measurement of proliferative activity, and that the growth fraction of colorectal cancers is a useful prognostic indicator. We suggested therefore that the poor survival of patients with p53-positive tumours might be associated with a high cell proliferative activity. From the findings, it may be concluded that the p53 immunoreactivity detected by use of PAb1801 is a useful prognostic indicator of colorectal cancers, and that it would permit the preoperative analysis of biopsy specimens.

### References

ARMITAGE, N.C., ROBINS, R.A., EVANS, D.F., TURNER, D.R., BALDWIN, R.W. & HARDCASTLE, J.D. (1985). The influence of tumour cell DNA abnormalities on survival in colorectal cancer. *Br. J. Surg.*, **72**, 828-830.

BANKS, L., MATLASHEWSKI, G. & CRAWFORD, L. (1986). Isolation of human p53 specific monoclonal antibodies and their use in the study of human p53 expression. *Europ. J. Biochem.*, **159**, 529-534.

CATTORETTI, G., RILKE, F., ANDREOLA, S., D'AMATO, L. & DELIA, D. (1988). p53 expression in breast cancer. *Int. J. Cancer*, **41**, 178-183.

CRAWFORD, L.V., PIM, D.C., GURNEY, E.G., GOODFELLOW, P. & TAYLOR-PAPADIMITRIOU, J. (1981). Detection of a common feature in several human tumor cell lines -a 53,000-dalton protein. *Proc. Natl Acad. Sci. USA*, **78**, 41-45.

CRAWFORD, L.V., PIM, D.C. & LAMB, P. (1984). The cellular protein p53 in human tumours. *Mol. Biol. Med.*, **2**, 261-272.

FEARON, E.R. & VOGELSTEIN, B. (1990). A genetic model for colorectal tumorigenesis. *Cell*, **61**, 759-767.

IGGO, R., GATTER, K., BARTEK, J., LANE, D. & HARRIS, A.L. (1990). Increased expression of mutant form of p53 oncogene in primary lung cancer. *Lancet*, **335**, 675-679.

IWAYA, K., TSUDA, H., HIRAIDE, H., TAMAKI, K., TAMAKUMA, S., FUKUTOMI, T., MUKAI, K. & HIROHASHI, S. (1991). Nuclear p53 immunoreaction associated with poor prognosis of breast cancer. *Jpn. J. Cancer Res.*, **82**, 835-840.

JAPANESE RESEARCH SOCIETY FOR CANCER OF THE COLON AND RECTUM. (1983). General rules for clinical and pathological studies on cancer of the colon, rectum and anus. *Jpn. J. Surg.*, **13**, 557-573.

KAWASAKI, Y., MONDEN, T., MORIMOTO, H., MUROTANI, M., MIYOSHI, Y., KOBAYASHI, T., SHIMANO, T. & MORI, T. (1992). Immunohistochemical study of p53 expression in microwave-fixed, paraffin-embedded sections of colorectal carcinoma and adenoma. *Am. J. Clin. Pathol.*, **97**, 244-249.

KERN, S.E., FEARON, E.R., TERSMETTE, K.W.F., ENTERLINE, J.P., LEPPERT, M., NAKAMURA, Y., WHITE, R., VOGELSTEIN, B. & HAMILTON, S.R. (1989). Allelic loss in colorectal carcinoma. *JAMA*, **261**, 3099-3103.

LANE, D.P. & CRAWFORD, L.V. (1979). T antigen is bound to a host protein in SV40-transformed cells. *Nature*, **278**, 261-263.

LANE, D.P. & BENCHIMOL, B. (1990). p53: oncogene or antioncogene? *Genes Dev.*, **4**, 1-8.

LEVINE, A.J. (1990). Tumor suppressor genes. *Bioassays*, **12**, 60-66.

MERCER, W.E., NELSON, D. DELEO, A.B., OLD, L.J. & BASERGA, R. (1982). Microinjection of monoclonal antibody to protein p53 inhibits serum-induced DNA synthesis in 3T3 cells. *Proc. Natl Acad. Sci. USA*, **79**, 6309-6312.

OREN, M., MALTZMAN, W. & LEVINE, A.J. (1981). Post-translational regulation of the 54K cellular tumor antigen in normal and transformed cells. *Mol. Cell Biol.*, **1**, 101-110.

PURDIE, C.A., O'GRADY, J., PIRIS, J., WYLLE, A.H. & BIRD, C.C. (1991). p53 expression in colorectal tumors. *Am. J. Pathol.*, **138**, 807-813.

REICH, N.C., OREN, M. & LEVINE, J. (1983). Two distinct mechanisms regulate the levels of a cellular tumor antigen, p53. *Mol. Cell Biol.*, **3**, 2143-2150.

REICH, N.C. & LEVINE, A.J. (1984). Growth regulation of a cellular tumour antigen, p53, in transformed cells. *Nature*, **308**, 199-201.

ROTTER, V., ABUTBUL, H. & BEN-ZE'EV. (1983). p53 transformation-related protein accumulates in the nucleus of transformed fibroblasts in association with the chromatin and is found in the cytoplasm of non-transformed fibroblasts. *EMBO J.*, **7**, 1041-1048.

SATO, Y., MUKAI, K., WATANABE, S., GOTO, M. & SHIMOSATO, Y. (1986). The AMeX method; a simplified technique of tissue processing and paraffin embedding with improved preservation of antigens for immunostaining. *Am. J. Pathol.*, **125**, 431-435.

SCOTT, N., SAGAR, P., STEWART, J., BLAIR, G.E., DIXON, M.F. & QUIRKE, P. (1991). p53 in colorectal cancer: clinicopathological correlation and prognostic significance. *Br. J. Cancer*, **63**, 317-319.

YAMAGUCHI, A., KUROSAKA, Y., FUSHIDA, S., KANNO, M., YONEMURA, Y., MIWA, K. & MIYAZAKI, I. (1992). Expression of p53 protein in colorectal cancer and its relationship to short-term prognosis. *Cancer*, **70**, 2778-2784.

WOLLEY, R.C., SCHREIBER, K., KOSS, L.G., KARAS, M. & SHERMAN, A. (1982). DNA distribution in human colon carcinomas and its relationship to clinical behaviour. *J. Natl Cancer Inst.*, **69**, 15-26.


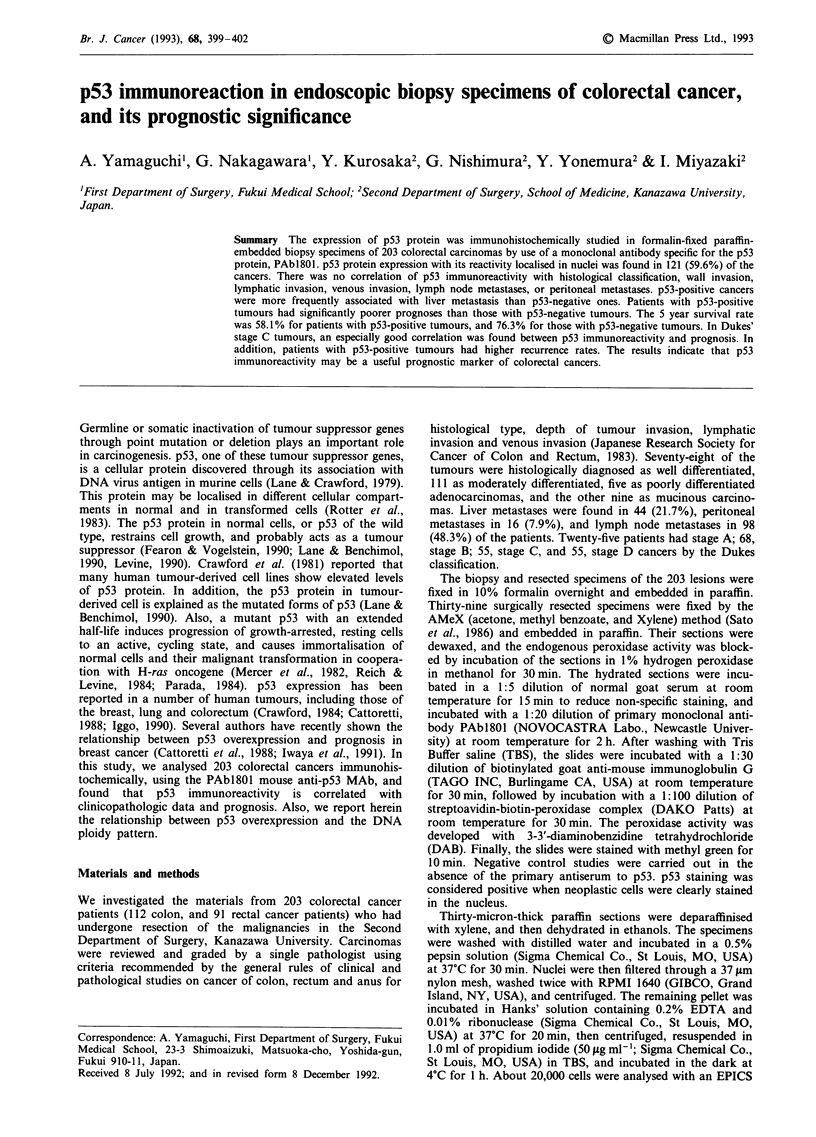

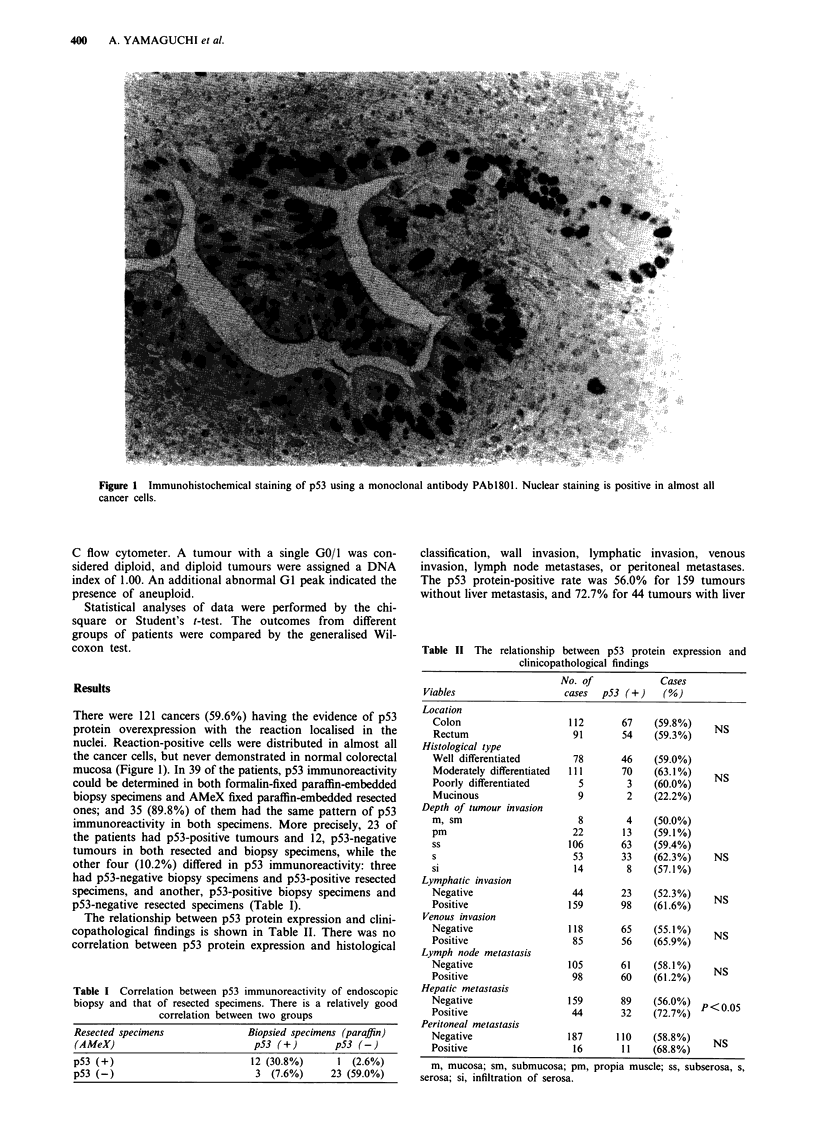

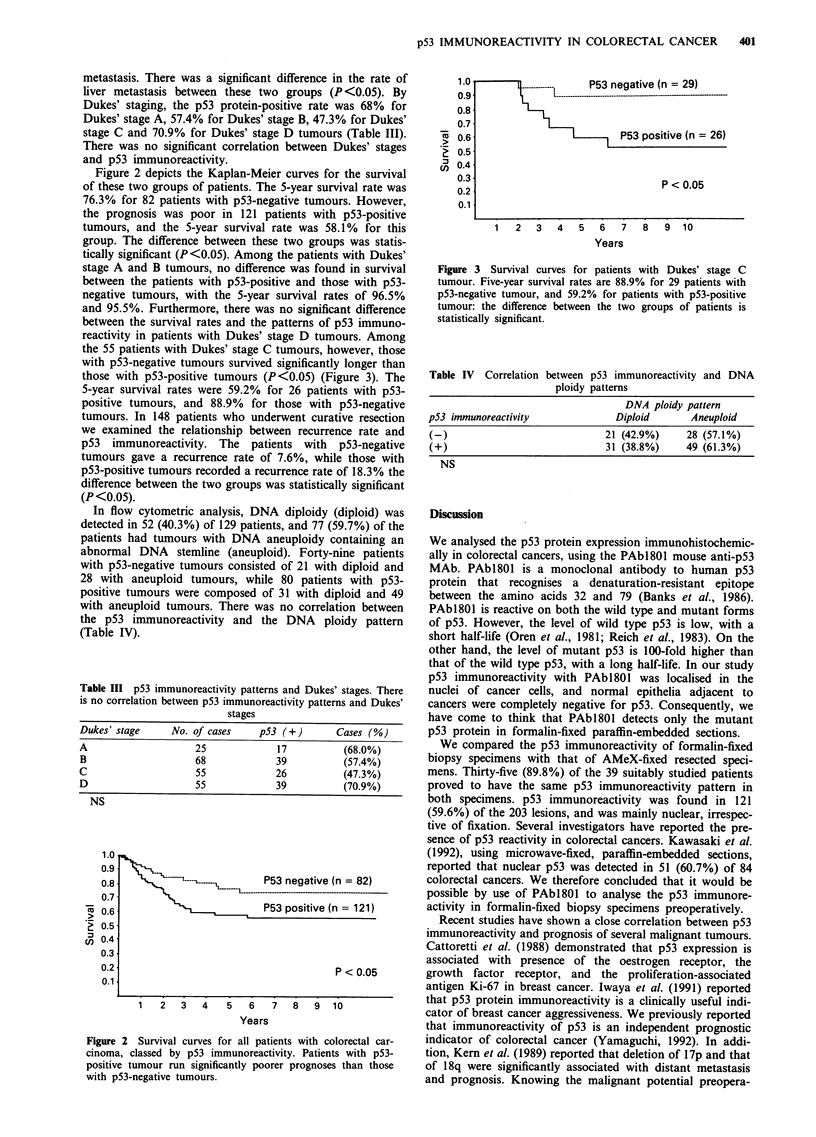

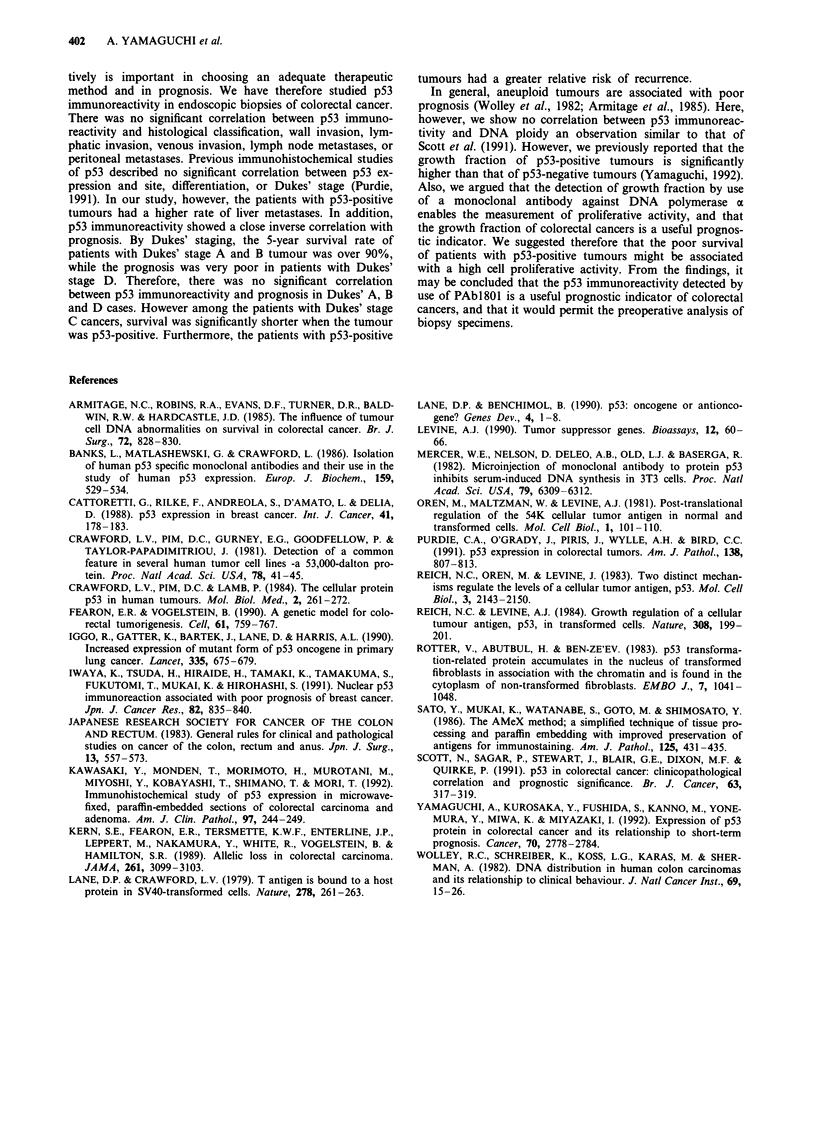


## References

[OCR_00457] Armitage N. C., Robins R. A., Evans D. F., Turner D. R., Baldwin R. W., Hardcastle J. D. (1985). The influence of tumour cell DNA abnormalities on survival in colorectal cancer.. Br J Surg.

[OCR_00461] Banks L., Matlashewski G., Crawford L. (1986). Isolation of human-p53-specific monoclonal antibodies and their use in the studies of human p53 expression.. Eur J Biochem.

[OCR_00467] Cattoretti G., Rilke F., Andreola S., D'Amato L., Delia D. (1988). P53 expression in breast cancer.. Int J Cancer.

[OCR_00472] Crawford L. V., Pim D. C., Gurney E. G., Goodfellow P., Taylor-Papadimitriou J. (1981). Detection of a common feature in several human tumor cell lines--a 53,000-dalton protein.. Proc Natl Acad Sci U S A.

[OCR_00478] Crawford L. V., Pim D. C., Lamb P. (1984). The cellular protein p53 in human tumours.. Mol Biol Med.

[OCR_00482] Fearon E. R., Vogelstein B. (1990). A genetic model for colorectal tumorigenesis.. Cell.

[OCR_00486] Iggo R., Gatter K., Bartek J., Lane D., Harris A. L. (1990). Increased expression of mutant forms of p53 oncogene in primary lung cancer.. Lancet.

[OCR_00491] Iwaya K., Tsuda H., Hiraide H., Tamaki K., Tamakuma S., Fukutomi T., Mukai K., Hirohashi S. (1991). Nuclear p53 immunoreaction associated with poor prognosis of breast cancer.. Jpn J Cancer Res.

[OCR_00503] Kawasaki Y., Monden T., Morimoto H., Murotani M., Miyoshi Y., Kobayashi T., Shimano T., Mori T. (1992). Immunohistochemical study of p53 expression in microwave-fixed, paraffin-embedded sections of colorectal carcinoma and adenoma.. Am J Clin Pathol.

[OCR_00510] Kern S. E., Fearon E. R., Tersmette K. W., Enterline J. P., Leppert M., Nakamura Y., White R., Vogelstein B., Hamilton S. R. (1989). Clinical and pathological associations with allelic loss in colorectal carcinoma [corrected].. JAMA.

[OCR_00516] Lane D. P., Crawford L. V. (1979). T antigen is bound to a host protein in SV40-transformed cells.. Nature.

[OCR_00524] Levine A. J. (1990). Tumor suppressor genes.. Bioessays.

[OCR_00528] Mercer W. E., Nelson D., DeLeo A. B., Old L. J., Baserga R. (1982). Microinjection of monoclonal antibody to protein p53 inhibits serum-induced DNA synthesis in 3T3 cells.. Proc Natl Acad Sci U S A.

[OCR_00534] Oren M., Maltzman W., Levine A. J. (1981). Post-translational regulation of the 54K cellular tumor antigen in normal and transformed cells.. Mol Cell Biol.

[OCR_00539] Purdie C. A., O'Grady J., Piris J., Wyllie A. H., Bird C. C. (1991). p53 expression in colorectal tumors.. Am J Pathol.

[OCR_00549] Reich N. C., Levine A. J. (1984). Growth regulation of a cellular tumour antigen, p53, in nontransformed cells.. Nature.

[OCR_00544] Reich N. C., Oren M., Levine A. J. (1983). Two distinct mechanisms regulate the levels of a cellular tumor antigen, p53.. Mol Cell Biol.

[OCR_00554] Rotter V., Abutbul H., Ben-Ze'ev A. (1983). P53 transformation-related protein accumulates in the nucleus of transformed fibroblasts in association with the chromatin and is found in the cytoplasm of non-transformed fibroblasts.. EMBO J.

[OCR_00561] Sato Y., Mukai K., Watanabe S., Goto M., Shimosato Y. (1986). The AMeX method. A simplified technique of tissue processing and paraffin embedding with improved preservation of antigens for immunostaining.. Am J Pathol.

[OCR_00567] Scott N., Sagar P., Stewart J., Blair G. E., Dixon M. F., Quirke P. (1991). p53 in colorectal cancer: clinicopathological correlation and prognostic significance.. Br J Cancer.

[OCR_00581] Wolley R. C., Schreiber K., Koss L. G., Karas M., Sherman A. (1982). DNA distribution in human colon carcinomas and its relationship to clinical behavior.. J Natl Cancer Inst.

[OCR_00575] Yamaguchi A., Kurosaka Y., Fushida S., Kanno M., Yonemura Y., Miwa K., Miyazaki I. (1992). Expression of p53 protein in colorectal cancer and its relationship to short-term prognosis.. Cancer.

